# Non-fluent agrammatic variant of primary progressive aphasia in a bilingual Mandarin/English speaker: a case report

**DOI:** 10.1093/arclin/acag027

**Published:** 2026-04-27

**Authors:** Belinda Y Zhang, Bailey L Ortiz, Shelley Peery

**Affiliations:** Department of Psychology, University of Southern California, Los Angeles, CA, USA; Department of Psychology, Palo Alto University, Palo Alto, CA, USA; San Francisco Neuropsychology PC, San Francisco, CA, USA

**Keywords:** frontotemporal dementia, aphasia, cross-cultural/minority, assessment, bilingualism, language and language disorders

## Abstract

**Objective:**

Non-fluent agrammatic variant of primary progressive aphasia (nfvPPA) is a neurodegenerative condition associated with effortful speech and agrammatism in language production. The neuropsychological assessment and diagnosis of nfvPPA in bilingual patients is not well characterized. This case report describes a 66-year-old bilingual Mandarin/English speaking male patient who experienced gradual progression of aphasia in both languages with initial preferential sparing of Mandarin.

**Method:**

The patient was seen for a comprehensive neuropsychological evaluation administered primarily in Mandarin Chinese with select subtests in English. Language functioning was assessed using the Bilingual Aphasia Screening Test.

**Results:**

Consistent with a diagnosis of nfvPPA, the patient’s neuropsychological assessment revealed agrammatism, apraxia of speech, and impaired comprehension of complex commands. Preserved language abilities included single-word comprehension, object knowledge, and lexical discrimination. In other domains, verbal and visual memory were impaired, while attention, processing speed, visuospatial skills, and motor functioning were preserved.

**Conclusion:**

This case illustrates a comprehensive neuropsychological battery to assess for nfvPPA in bilingual patients. This case also has implications for understanding the clinical presentation and progression of symptoms for bilingual patients, highlighting the complex interaction between neurodegeneration and bicultural language representation.

## Introduction

Primary progressive aphasia (PPA) is a neurodegenerative condition characterized by the progressive and selective impairment of language function, with relative preservation of other cognitive abilities during the initial disease stages (Gorno-Tempini et al., 2011). Unlike aphasias caused by acute brain injury (e.g., stroke or trauma), which present abruptly and may feature focal cortical atrophy secondary to the injury, PPA develops gradually and is driven by an underlying neurodegenerative process that produces progressive atrophy of language-related cortical regions. PPA is classified under the umbrella of frontotemporal lobar degeneration (FTLD) and is most commonly associated with underlying tauopathy or TDP-43 proteinopathy, though Alzheimer’s pathology may also be present in some variants (Grossman, 2012a). To our knowledge, no studies have comprehensively characterized the global epidemiology of PPA. However, a recent registry study from Olmsted County, Minnesota reported an incidence of 0.14 per 100,000 person-years for non-fluent agrammatic variant of primary progressive aphasia (nfvPPA; Turcano et al., 2024), and a population-based study in France estimated an incidence of 1.14 per 100,000 person-years for PPA overall–substantially lower than the incidence rate of Alzheimer’s disease (35.7 per 100,000 person-years; Mouton et al., 2022).

There are three clinical variants of PPA, each with distinct linguistic, anatomical, and pathological features: the semantic variant (svPPA), the logopenic variant (lvPPA), and the nfvPPA. In svPPA, patients demonstrate object knowledge loss and impaired single-word comprehension, with characteristic atrophy in the left anterior temporal lobe (Collins et al., 2016; Hodges et al., 1992; Montembeault et al., 2018). LvPPA presents with impaired single-word retrieval and sentence repetition, but relatively intact grammar and motor speech (Gorno-Tempini et al., 2004; Montembeault et al., 2018; Tippett & Keser, 2022). LvPPA is associated with posterior perisylvian and parietal atrophy and often linked to underlying Alzheimer’s disease pathology (Mandelli et al., 2023; Preiß et al., 2019).

NfvPPA is characterized by agrammatic language production and/or apraxia of speech, resulting in halting, effortful, and grammatically impoverished output (Grossman, 2012b; Montembeault et al., 2018). Comprehension of complex syntax may also be affected, although single-word comprehension and object knowledge are typically spared early in the disease course (Gorno-Tempini et al., 2011; Mesulam, 2001). Structural imaging often reveals asymmetric atrophy in the left posterior fronto-insular region, particularly involving the inferior frontal gyrus (i.e., Broca’s area), supplementary motor area, and anterior insula (Gorno-Tempini et al., 2011; Grossman, 2012b; Josephs et al., 2006). Functional imaging and post-mortem studies often reveal tau or TDP-43 inclusions in these regions (Grossman, 2012a).

Although language impairment is the defining feature of PPA, patients often experience broader cognitive, social, and emotional consequences over time. In nfvPPA, speech difficulties can severely affect verbal expression, leading to social withdrawal, decreased quality of life, and increased caregiver burden (Modirrousta et al., 2013). Accurate diagnosis, particularly in early stages, relies on comprehensive neuropsychological assessment tailored to the individual’s language and cultural background.

Neuropsychological assessments for patients presenting with language symptoms become particularly complex in bilingual individuals, who make up a significant portion of the global population (Rivera Mindt et al., 2008). Bilingualism introduces variability in expression of language-related neurodegenerative disorders due to differences in language dominance, age of acquisition, proficiency, and daily usage patterns (Anderson et al., 2017). Research suggests that the first-acquired language may be more resistant to neurodegeneration, while the second-acquired language is often more vulnerable, especially when acquired later or used less frequently (Costa et al., 2019). Consequently, bilingual patients may present with asymmetric language impairment and often revert to more frequent use of their first-acquired language as the disease progresses (Mendez et al., 1999).

The neural organization of linguistic processing is shaped by the structural properties of each language. Mandarin Chinese is a logographic, tonal language with distinct phonological, syntactic, and orthographic features compared to alphabetic languages such as English (Cao et al., 2011; Ling & Grüter, 2022; Wang et al., 2015; Zhang & Xing, 2023). For example, Mandarin employs monosyllabic morphemes, lacks verb conjugation, and relies heavily on word order and context for meaning (Packard, 2000). Neuroimaging studies indicate that reading and writing Chinese engage a broader bilateral network, with greater right hemisphere activation, compared to English (Qi et al., 2015; Tan et al., 2005). These linguistic differences may influence the manifestation of aphasia symptoms and should be considered when evaluating bilingual patients whose first-acquired language is Chinese (Tee et al., 2022), as they may exhibit language-specific error patterns (e.g., homophone substitutions, orthographically similar errors, and compound word errors) that differ from those observed in alphabetic languages.

Despite increasing recognition of the importance of linguistic diversity in neurodegenerative disease, very few published reports have examined how nfvPPA presents in Mandarin-speaking bilingual individuals. A better understanding of bilingual PPA presentations is essential for ensuring equitable access to accurate diagnosis, appropriate neuropsychological assessment, and culturally sensitive care.

In this case report, we describe a 66-year-old, right-handed, bilingual Mandarin-English speaking man who presented with a 2-year history of gradually progressive speech and language difficulties. Notably, his Mandarin—his first acquired and more dominant language—remained relatively preserved early in the disease course, while his English—acquired later in life—showed more pronounced deterioration. This case illustrates the cognitive profile of nfvPPA in a bilingual individual with an orthographic first-acquired language and highlights the importance of culturally responsive assessment practices in the evaluation of language-based neurodegenerative conditions.

## Materials and methods

The patient and his family provided informed consent for participation in this case study and publication of findings. The patient was referred by a neurologist at a large private hospital system and completed a comprehensive 7-h neuropsychological evaluation in a private practice setting in the San Francisco Bay Area. The evaluation was conducted by a bilingual (Mandarin/English) clinical psychology graduate student (B.Y.Z.) under the supervision of a licensed clinical neuropsychologist (S.P.). Because the examiner was fluent in both Mandarin and English, all testing and interviews were conducted directly by the examiner without the use of interpretive services.

The evaluation consisted of a clinical interview with the patient in Mandarin and a collateral interview with his adult daughter in English to gather information on symptom onset, progression, medical and psychosocial history, and current level of functioning. Although the patient was able to participate in the interview and respond to simple questions, his expressive language impairment limited his ability to provide detailed or elaborated responses. Testing was conducted in both Mandarin and English, based on the patient’s proficiency and clinical presentation.

A neuropsychological battery was selected to assess multiple domains of cognitive functioning: estimated pre-morbid intellectual ability, attention and working memory, processing speed, executive function, verbal and visual episodic memory, visuospatial ability, and language. Test instruments were selected to maximize cross-linguistic validity and accommodate the patient’s linguistic and cultural background. Culturally appropriate measures were used whenever possible. Behavioral inventories and mood questionnaires were administered to assess emotional and psychosocial functioning. Self-report questionnaires were administered orally by the examiner and interpreted into Mandarin to ensure comprehension and accurate responding. Performance validity was evaluated through clinical observation of test engagement and behavior, as formal validity measures were not administered due to the severity of the patient’s deficits. Neuropsychological test performance is summarized in Table 1. Neuropsychological instruments used, administration language, and normative references are provided in the Supplementary material.

**Table 1:** Raw scores, percentiles, and qualitative interpretations for neuropsychological assessment measures

Measure	Raw score	Percentile	Interpretation
**Global cognition/intellectual functioning**			
MoCA Beijing^a^	4	—	Severe cognitive impairment
WAIS-IV Chinese^b^			
Similarities	2	1	Exceptionally low
Matrix reasoning	2	<1	Exceptionally low
Digit span	6	<1	Exceptionally low
Coding	40	25	Average
Block design	28	37	Average
Block design (w/o time bonus)	28	37	Average
Symbol search	6	2.3	Below average
Figure weights	5	5	Exceptionally low
Picture completion	4	25	Average
Visual puzzles	8	25	Average
Estimated FSIQ^c^	—	6	Below average
PRI^d^	—	25	Average
PSI^e^	—	8	Below average
**Attention and working memory**			
WAIS-IV Chinese^b^			
Digit span forward	3	<1	Exceptionally low
Digit span backward	2	5	Below average
Digit span sequencing	1	<1	Exceptionally low
WMS-III spatial span^f^			
Spatial span forward	5	9	Low average
Spatial span backward	5	25	Average
Total	10	16	Low average
**Language functioning**			
WAIS-IV Chinese^b^			
Similarities	2	1	Exceptionally low
BDAE^g^			
Non-verbal agility	11/12	—	Within normal limits
Verbal agility	6/14	—	Impaired
BAT^h^			
Pointing	9/10	—	Within normal limits
Simple and semi-complex commands	8/10	—	Within normal limits
Complex commands	0/5	—	Impaired
Repetition of sentences	1/7	—	Impaired
Series (Mandarin)	1/3	—	Impaired
Series (English)	0/3	—	Impaired
Phonemic Fluency	—	—	Discontinued/impaired
Category Fluency	7	—	Impaired
Repetition of words and nonsense words	30/30	—	Within normal limits
Lexical discrimination	25/30	—	Within normal limits
Pyramids and palm trees	36/52	—	Within normal limits
**Memory functioning**			
FOME^i^			
Trial 1	2	—	—
Trial 2	3	—	—
Trial 3	4	—	—
Trial 4	1	—	—
Trial 5	0	—	—
Total storage	10	—	—
Delayed recall	0	<1	Exceptionally low
Recognition	9	—	—

(*Continued*)

**Table 1:** Continued

Measure	Raw score	Percentile	Interpretation
BVMT-R^j^			
Trial 1	1	2	Below average
Trial 2	0	<1	Exceptionally low
Trial 3	1	<11	Exceptionally low
Total recall	2	<1	Exceptionally low
Learning	0	2	Below average
Delayed recall	0	<1	Exceptionally low
Percent retained	0%	<1	Exceptionally low
Recognition hits	6	>15	Within normal limits
Recognition false alarms	1	6–10	Low average
Recognition discrimination index	5	11–16	Low average
Recognition Response Bias	0.75	3–5	Below average
Copy	12	—	Within normal limits
**Executive functioning**			
WAIS-IV Chinese^b^			
Matrix reasoning	2	<1	Exceptionally low
Similarities	2	1	Exceptionally low
Color trails test			
Color trails 1	182 sec	<1	Exceptionally low
Prompts	6	—	—
Errors	0	62	Average
Color Trails 2	263 sec	<1	Exceptionally low
Prompts	10	—	—
Errors	0	31	Average
Behavioral Dyscontrol Scale	10/19	—	Below expectation
Ramparts	—	—	No errors
Spirals	—	—	7 Perseverative errors
Ms and Ns	—	—	2 Errors
**Visuospatial functioning**			
WAIS-IV Chinese^b^			
Matrix reasoning	2	<1	Exceptionally low
Visual puzzles	8	25	Average
BVMT-R copy^j^	12	—	Within normal limits
**Motor Functioning**			
Grooved pegboard			
Dominant hand	129 s	8	Below average
Non-dominant hand	88 s	63	Average
Praxis screen	18/18	—	Within normal limits

^a^Montreal cognitive assessment, Beijing version.

^b^Chinese edition of Wechsler Adult Intelligence Scale-Fourth Edition. The Chinese version of the WAIS-IV was standardized on stratified samples reflecting population demographics (e.g., age and education) across urban and rural regions in China (Cui et al., 2017), supporting its use for this Mandarin-dominant patient, although his extended residence in the United States may limit full representativeness.

^c^Full Scale Intelligence Quotient on the WAIS-IV.

^d^Perceptual Reasoning Index on the WAIS-IV.

^e^Processing Speed Index on the WAIS-IV.

^f^Wechsler Memory Scale-III Chinese Edition.

^g^Boston Diagnostic Aphasia Examination.

^h^Bilingual Aphasia Test.

^i^Fuld Object-Memory Evaluation.

^j^Brief Visuospatial Memory Test-Revised.

## Results

### Social history

The patient was born and raised in Taiwan and is a native Mandarin speaker. He had standard English classes during his schooling in Taiwan, though the precise age of onset and quality of instruction could not be determined. Based on this information, English is considered a later-acquired second language for this patient. He earned a bachelor’s degree in international business in Taiwan, which included coursework in both Mandarin and English, and immigrated to the United States as a young adult. He denied any learning or behavioral difficulties in school. He worked in a business operations role at a technology company for several decades in the United States and retired in his early 60s, reportedly unrelated to cognitive changes. Details regarding specific job duties or the extent of speech use in his work are not available. He has been married for several decades and has adult children. He reported having strong social support, including his immediate and extended family, many of whom live nearby.

### History of presenting problem

The patient expressed some awareness of his language difficulties but was unable to elaborate on the nature of his concerns. As such, most of the clinical history was obtained from his daughter, supplemented by review of medical records. She reported that he has had progressive language decline with insidious onset, initially in his second language (English) and later in his native language (Mandarin). His language impairments may have begun subtly as early as 6 years prior to the current evaluation. For example, beginning at around age 60 years, he was unable to schedule house-related tasks due to difficulty communicating with various handypersons in English, although the nature of his language difficulties was unclear. His daughter reported that his language symptoms became more prominent approximately 2 years prior to the evaluation, including decreased speech output and using shorter, telegraphic phrases or single words to communicate. Additionally, he had worsening ability to speak and understand English, despite having used professional English at work for several decades, and would often revert to Mandarin. Additional changes in his language included using formal vocabulary in Mandarin, even when speaking to close friends or family members. For example, he referred to his daughter using the formal pronoun “您  (nín)” for “you” instead of the culturally typical informal “你 (nǐ).” In Mandarin, it is customary (Tee et al., 2022) for elders to use informal language when speaking to younger family members (Gu, 1990; Yu, 2002); thus, his formal language use was atypical and stood out to his daughter.

In addition to language impairments, the patient’s daughter reported the emergence of memory difficulties following the onset of his language symptoms, as well as significant mood changes after his initial language and speech decline. He developed new-onset depressed mood, anxiety, and decreased energy approximately 2 years prior to the evaluation, although these symptoms have improved over the past 6 months to 1 year.

### Current complaints

The patient and his daughter reported difficulties primarily with speech and language, including worsening expressive and receptive language in both his native language (Mandarin) and second language (English). He reported that he was proficient in English for several decades but now has near complete loss of ability to speak, read, or understand English. His daughter also reported prominent difficulties with speech output in Mandarin, along with some difficulty with comprehension of complex instructions.

Hallucinations, delusions, and suicidal ideation were denied. Appetite and sleep were stable. Vision and hearing were within normal limits. Falls or other motor changes were denied.

### Functional status

The patient manages his basic activities of daily living (ADLs) independently but requires assistance from family members for his instrumental ADLs, including bill payments, medical appointments, and grocery shopping. Additionally, the patient and his daughter completed inventories assessing his behavioral and emotional functioning. On the Patient Competency Rating Scale (Prigatano et al., 1986), a self-report rating of his level of independence in performing basic and instrumental activities of daily living, he endorsed greatest difficulty with communication, followed by travel and household care. His daughter completed the Informant Questionnaire on Cognitive Decline in the Elderly (Jorm, 1994) for longitudinal changes in cognition, indicating an overall decline in his cognitive functioning consistent with dementia.

### Medical and family history

Medical history included hypertension and benign prostatic hyperplasia. Family history was unremarkable; there was no known history of neurodegenerative conditions or other neurological disorders. Neuroimaging was acquired 2 years prior to the present evaluation. Per radiology report, magnetic resonance imaging of the brain indicated no acute infarct or hemorrhage; white matter chronic small vessel ischemic changes were present. F-18 fluorodeoxyglucose positron emission tomography, per radiology report, indicated normal biodistribution for stated age bracket and cerebral volume. Computed tomography (CT) of the head with no contrast was within normal limits.

### Behavioral observations

The patient presented as cooperative, socially appropriate, and engaged throughout the evaluation. He was right-handed, ambulated independently, and appeared well-groomed. A mild action tremor was noted in his dominant right hand during writing tasks. His conversational speech in Mandarin was telegraphic and halting, with marked word-finding difficulties. Although his speech was not off-topic, contextual cues were often required to interpret his intended meaning. Comprehension of short, simple phrases appeared intact. His affect was euthymic and at times appeared euphoric despite evident challenges, suggesting reduced awareness of deficits.

### Test results

Where available, results were normed using Chinese standardized samples and corrected for age and education. United States-based norms were used in the absence of Chinese normative data. Descriptors follow the American Academy of Clinical Neuropsychology (AACN) consensus guidelines (Guilmette et al., 2020). Due to the limited availability of validated tools for Mandarin-English bilingual Americans, some test scores may underestimate his true abilities; results were interpreted with caution and cultural context in mind.

#### Intellectual functioning

WAIS-IV Chinese results indicated a discrepant profile. Verbal reasoning and non-verbal reasoning were *Exceptionally Low*, suggesting profound impairments in abstract reasoning. Processing speed tasks were in the *Below Average* to *Average* range, indicating relative preservation of simple visual scanning and motor speed. The patient’s estimated Full-Scale IQ was in the *Below Average* range, although this likely underestimates his premorbid functioning given significant language difficulties and disease progression.

#### Attention and working memory

Digit Span performance was *Exceptionally Low*, particularly for forward and sequencing tasks, but this is likely confounded by language output and auditory-verbal working memory demands. Nonverbal attention measures (i.e., Spatial Span) were in the *Low Average* to *Average* range, suggesting that core attentional capacity was intact once linguistic demands were minimized.

#### Language functioning

Comprehensive language testing revealed markedly effortful, halting speech and reduced grammatical complexity. The patient demonstrated expressive deficits across both Mandarin and English, though impairments were more pronounced in English. The following sections describe the patient’s language performance separately in Mandarin and English.

##### Mandarin language

On Mandarin language testing, the patient had intact object knowledge, single-word comprehension, and single-word repetition. He correctly identified objects by pointing with 90% accuracy. He additionally was able to name 8 out of 10 objects on a confrontation naming screener (Fuld Object-Memory Evaluation [FOME]), performing within expectation. Single-step and simple two-step commands were completed with 80% accuracy (BAT Simple & Semi-Complex Commands = 8/10), but complex commands were impaired (BAT Complex Commands = 0/5). Repetition of single-character and two-character words was preserved (BAT Repetition of Words and Nonsense Words = 30/30), whereas repetition of short sentences was impaired (BAT Repetition of Sentences = 1/7). Lexical discrimination of real versus nonsense words was 83% accurate (BAT Lexical Discrimination = 25/30).

##### Writing/orthographic errors in Mandarin

**Figure 1:**
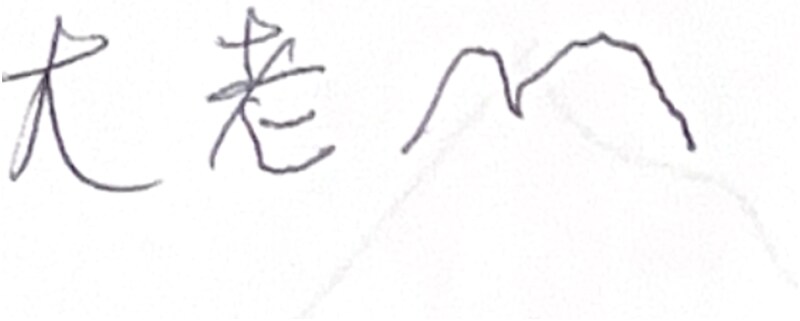
Sample of spontaneous written Chinese characters produced by the patient during a visuospatial memory test.

On the delayed recall trial of the Brief Visuospatial Memory Test (BVMT), when asked to recall the visual figures, he instead produced the Chinese characters **大老** (dà lǎo). This likely reflected an intended phrase of **太老了** (dà lǎo le; “too old”), a self-deprecating comment about memory (see Fig. 1). The response illustrates (1) radical omission/substitution – the small dot (丶) distinguishing **太** from **大** was absent, and (2) grammatical particle omission, as the low-complexity but obligatory particle **了** was not produced.

##### Picture description task in Mandarin

During the Cookie Theft picture description in Mandarin, the patient produced short, halting utterances rich in content words but lacking grammatical morphology. A verbatim excerpt and translation are provided in Table 2.

**Table 2:** Verbatim Mandarin speech sample from the Cookie Theft picture description task, with English translation

Mandarin (verbatim)	English translation
妈妈。。妹妹。。哥哥。。算了。。妈妈在厨房。。妈妈不小心把。。那个。。在厨房里把水打到地上。。老大不管妈妈和爸爸自己买一个糖爬到树上。。妹妹跟妈妈不管事就是 *(zhuang yang)* 她的喜欢吃的糖	Mother. . . sister. . . brother. . . never mind. . . mother in the kitchen. . . mother accidentally. . . that. . . spilled water on the floor in the kitchen. . . the older one doesn’t care about mother and father, buys a candy, climbs up the tree. . . sister is not paying attention to mother. . . just (pretends) the candy she likes to eat.

The sample contains multiple features characteristic of nfvPPA, including (1) agrammatism, with omission of aspect markers (e.g., **了** *le)*, structural particles (e.g., **的**  *de*), and classifiers; (2) telegraphic syntax, consisting largely of noun-verb sequences with frequent topic restarts; (3) phonological/tonal distortion, as in the non-standard form *“zhuang yang,”* which approximates **装样子** (*zhuāng yàngzi*, “pretend”) but omits the final syllable **子**  *(zi)* and neutralizes the fourth tone of **样** (*yang)*. Content words such as **妈妈**  *(mother),*  **水**  *(water),*  **糖**  * (candy)*, and **树**  *(tree)* were appropriately retrieved, demonstrating preserved lexical-semantic knowledge despite profound deficits in grammar and motor speech.

##### English language

Despite using professional English at work for several decades, the patient reported a near complete loss of ability to speak and understand English. On the Bilingual Aphasia Test (BAT; Paradis & Libben, 1987), the patient was asked about his English background. He reported that his pre-morbid speaking and reading ability was proficient (i.e., 4 on a scale from 1 to 5), while his writing ability was mediocre (i.e., 3 on a scale from 1 to 5) and spoke English at work, with friends, and at home with his children. Prior to symptom onset, he reported that he spoke, read, and wrote using English daily. On the BAT (English Version), he could not complete any items on Series (e.g., naming days of the week, counting from 1 to 25, naming all the months of the years). On the Boston Diagnostic Aphasia Exam (BDAE; Roth, 2011), his verbal agility was impaired (6/14); he was able to repeat simple words multiple times (e.g., mama. . . mama. . ., thanks. . . thanks.) but could not repeat longer words more than once (e.g., caterpillar, baseball player). He declined additional English testing, citing fatigue, and frustration with his limited English output.

#### Memory functioning

Both verbal and non-verbal memory scores were impaired for learning and delayed recall, with recognition memory relatively preserved. On the FOME (Fuld, 1981), which requires tactile identification of objects followed by verbal recall, responses were provided in Mandarin. Given relatively preserved recognition, impaired free recall may partly reflect difficulty expressing retrieved words, although both expressive language and memory factors likely contributed.

#### Executive functioning

**Figure 2:**
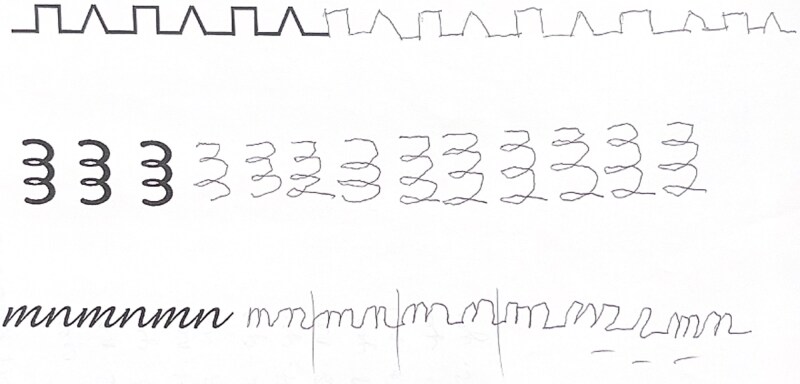
Patient production of graphical or alternating sequences, including the “ramparts” design, spirals, and Ms and Ns.

Marked deficits were observed in visuomotor set-shifting and abstract reasoning. Behavioral measures (BDS = 10/19) and qualitative observations (e.g., preservative errors on graphical or alternating sequences; see Fig. 2) indicate dysexecutive features, including reduced cognitive flexibility and poor inhibitory control. These findings are consistent with frontal system involvement.

#### Visuospatial and motor functioning

Performances on Block Design, Visual Puzzles, and BVMT Copy were *Average*, suggesting intact visuospatial construction and visual-perceptual skills. Fine motor coordination was slower in the dominant hand (R) but intact in the non-dominant hand (L), suggesting asymmetric motor slowing often seen in left-hemisphere neurodegenerative syndromes. Although an action tremor of the right hand was noted in the medical chart, no tremor was observed during the current evaluation. No dyspraxia was observed on a screener.

### Clinical diagnosis

Overall, the patient’s cognitive profile is characterized by prominent motor speech impairments and agrammatism, which preceded other cognitive symptoms. Therefore, his clinical syndrome was most consistent with nfvPPA. Given that the patient requires assistance with his instrumental activities of daily living and has decline across multiple cognitive domains, he met criteria for a major neurocognitive disorder (i.e., dementia).

## Discussion

This case study offers a detailed characterization of nfvPPA in a Mandarin-English bilingual speaker, assessed comprehensively in his primary language (Mandarin). The evaluation revealed classic features of nfvPPA, including agrammatism, apraxia of speech, and impaired comprehension of complex sentences with spared single-word comprehension and spared object knowledge.

The patient’s history of symptom onset further supports the diagnosis, with an insidious onset of motor speech impairments that preceded his decline in memory. Attention, processing speed, and visuospatial abilities were relatively preserved. Taken together, the patient’s constellation of symptoms is consistent with a clinical diagnosis of nfvPPA (Gorno-Tempini et al., 2011), which is characterized by early deficits in language production and grammar with relative sparing of other cognitive domains. Although involvement of additional cognitive domains in this patient suggests progression beyond the earliest stage of disease, the initial language impairments remain characteristic of nfvPPA.

From a motor standpoint, the patient exhibited slowed fine motor coordination in his dominant right hand. This asymmetry supports left-hemisphere dysfunction, consistent with the anatomical underpinnings of nfvPPA, which typically involves the left posterior fronto-insular region (Grossman, 2012c; Montembeault et al., 2018). Although lateralization was not clearly evident on neuroimaging, the clinical findings suggest predominant left-hemispheric involvement with primary impact on language.

A notable aspect of this case is the patient’s profound loss of English, despite decades of professional use. His near-complete inability to understand or speak English at the time of testing, relative to preserved Mandarin single-word knowledge and command-following, is consistent with prior observations that a less dominant or less frequently used language is often more vulnerable in bilingual cases of PPA (Karpathiou et al., 2018; Larner, 2012). This pattern aligns with theories of language attrition in bilingual aphasia (Fabbro, 2001), suggesting that both language proficiency and usage patterns modulate the course of neurodegeneration. In this case, Mandarin remained his first-acquired and socially dominant language and appeared more resilient as the disease progressed.

The relative preservation of the patient’s dominant language alongside disproportionate loss of his second language may reflect differences in the neural representation of first- versus second-acquired languages. Dominant languages are supported by extensive bilateral perisylvian networks with strong left-hemisphere involvement, whereas second languages, particularly those acquired later, recruit additional regions such as the dorsolateral prefrontal cortex and anterior cingulate to support greater cognitive control (Abutalebi, 2008; Kim et al., 1997; Perani et al., 2003; Sebastian et al., 2011). In nfvPPA, which affects left posterior fronto-insular regions critical for motor speech and grammar, this may contribute to disproportionate loss of English relative to Mandarin. Although Mandarin engages more bilateral networks, left-hemisphere dysfunction may disrupt speech production and syntax, while right-hemisphere regions supporting semantic and orthographic processing may initially compensate, preserving single-word comprehension.

A particularly striking feature of the patient’s expressive language was his use of the formal second-person pronoun “**您**  (nín)” for “you” rather than the expected informal “**你** (nǐ)” when speaking to his daughter. In Mandarin-speaking cultures, elder family members typically speak informally to younger relatives, reflecting hierarchical norms that emphasize age-based seniority in interpersonal communication (Gu, 1990; Yu, 2002), and his daughter noted that this formal address was a new and unusual shift from his baseline. This finding may reflect subtle changes in pragmatic language use or disruptions in social cognition (Harris et al., 2016; Kamath et al., 2019; Landin-Romero et al., 2024; Rousseaux et al., 2010; Shany-Ur & Rankin, 2011). It could also indicate reliance on more automatic or overlearned forms of speech as a compensatory strategy in the face of expressive language decline (Kempler & Goral, 2008; Rezaii et al., 2022; Zimmerer et al., 2020). Alternatively, it may suggest altered self-other mapping or a loss of contextual language flexibility, both of which have been associated with degeneration in frontal and insular networks implicated in nfvPPA (Botha et al., 2015; Gorno-Tempini et al., 2011; Rankin et al., 2006; Reyes et al., 2019). This unexpected sociolinguistic behavior underscores how culturally embedded linguistic patterns can serve as sensitive markers of neurodegenerative change.

In addition to factors such as pragmatics, social cognition, and reliance on overlearned language forms, the patient’s use of formal pronouns and atypical sentence structures in Mandarin may reflect underlying grammatical impairments characteristic of nfvPPA. Specifically, omission of obligatory aspect markers, structural particles, and telegraphic syntax suggest that these changes are not purely pragmatic or social, but also indicative of agrammatic processing deficits in Mandarin.

Psychologically, the patient’s emotional trajectory was notable. According to his daughter, the early phase of disease was marked by confusion, anxiety, and tearfulness—symptoms common in individuals confronting initial loss of communicative competence. However, by the time of the neuropsychological evaluation, the patient presented at times as euphoric, with minimal insight into his impairments. The shift likely reflects emerging anosognosia, which is increasingly recognized in neurodegenerative diseases and may indicate progression of disease into networks involved in self-awareness and metacognition (Hallam et al., 2020; Rosen et al., 2010; Sunderaraman & Cosentino, 2017).

Given the presence of memory and executive impairments at the time of evaluation, alternative etiologies including Alzheimer’s disease (AD), svPPA, and lvPPA were carefully considered. However, several aspects of the clinical course and qualitative language profile argue against these diagnoses as the primary syndrome. AD was considered less likely given the insidious onset of motor speech and grammatical impairments that clearly preceded memory difficulties by several years, which is atypical for AD, where episodic memory impairment is usually an early and defining feature (McKhann et al., 2012). Additionally, the patient’s memory profile, impaired free recall with relatively preserved recognition, suggests a retrieval-based deficit consistent with frontal system involvement rather than the encoding and storage deficits characteristic of AD. These deficits likely reflect progression of nfvPPA into frontal/executive networks rather than an alternate primary etiology. Preserved visuospatial abilities, attention, and processing speed further reduce the likelihood of an AD-related language presentation. SvPPA was also considered unlikely. Despite progressive language impairment, the patient demonstrated preserved single-word comprehension, intact object knowledge, and accurate lexical discrimination in Mandarin, which are inconsistent with the core semantic degradation of svPPA (Gorno-Tempini et al., 2011; Montembeault et al., 2018). Moreover, his speech was characterized by agrammatism and motor speech impairment rather than fluent but empty output. LvPPA was also evaluated; however, the patient did not exhibit its defining features, such as relatively preserved grammar and motor speech in the context of prominent word-finding pauses and phonological working memory-driven repetition deficits (Gorno-Tempini et al., 2011). Instead, his speech was effortful and halting, with clear agrammatism and apraxia of speech across both languages. Although sentence repetition was impaired, errors reflected grammatical simplification, omissions, and motor speech breakdown rather than phonological paraphasias or length-dependent effects typical of lvPPA, indicating a motor-grammatical deficit rather than a primary phonological mechanism. Taken together, the exclusion of these alternative diagnoses further supports nfvPPA as the most parsimonious clinical diagnosis.

There are several limitations of this study to consider. The clinical diagnosis could not be confirmed through neuroimaging or biomarker studies. Thus, while the profile is highly consistent with the clinical features of nfvPPA, alternative or co-occurring pathologies cannot be ruled out. However, this case highlights the value of comprehensive neuropsychological assessment in supporting a syndromic diagnosis when biomarker or imaging evidence is unavailable or inconclusive. Additionally, while the assessment included extensive oral language and cognitive testing, it did not formally evaluate written language. This is an area for future exploration, particularly in tonal or logographic languages such as Chinese. Prior research has shown that Chinese speakers with nfvPPA often exhibit specific writing impairments, including radical substitution and errors in compound character formation (Tee et al., 2022), which may help distinguish between PPA subtypes and offer insight into orthographic processing across languages. Furthermore, while we were able to establish an overall timeline of symptom progression, the available medical records and collateral history did not provide sufficient detail to specify the exact timing and characterization of his early communication difficulties and need for instrumental ADL assistance.

To date, most research and clinical tools for diagnosing PPA have been developed in monolingual English-speaking populations, leaving a critical gap in care for the growing number of bilingual and multilingual individuals worldwide. The present case demonstrates the feasibility and diagnostic value of conducting neuropsychological assessment in a patient’s dominant language using culturally and linguistically adapted tools. Without this adaptation, the extent of the patient’s preserved abilities may have been underestimated or mischaracterized.

Furthermore, this case supports the need for more systematic study on how language structure (e.g., tonal vs. non-tonal, logographic vs. alphabetic) interacts with neurodegenerative patterns. For example, Chinese syntax and morphology differ markedly from Indo-European languages (Alderete & O’Séaghdha, 2022; Banfi & Arcodia, 2009; Rosen et al., 2010), and it remains a question how these features modulate the expression and progression of agrammatism or apraxia of speech. Similarly, cultural factors such as the use of formal address terms and indirect communication styles may shape the presentation of pragmatic impairments in ways not well-captured by Western diagnostic norms.

In sum, this case adds to the literature on PPA in bilingual individuals and underscores the importance of linguistically and culturally appropriate assessment in the accurate diagnosis and management of neurodegenerative disease. It also highlights the resilience of deeply ingrained social and linguistic behaviors, even amid substantial language and cognitive decline, offering new directions for understanding preserved capacities in bilingual patients with PPA.

## Supplementary Material

acag027_nfvppa_supplementarymaterials_3_17_26
