# Supplementary material for: Non-fluent agrammatic variant of primary progressive aphasia in a bilingual Mandarin/English speaker: a case report
Source: Arch Clin Neuropsychol. 2026 Apr 27;41(3):acag027. doi: 10.1093/arclin/acag027 (PMC13112198; doi:10.1093/arclin/acag027)
Supplement: acag027_nfvppa_supplementarymaterials_3_17_26 [file acag027_nfvppa_supplementarymaterials_3_17_26.docx]

**Table S1.** Neuropsychological instruments, administration language, and normative references.

| **Instrument** | **Domain** | **Administration Language** | **Reference** |
| --- | --- | --- | --- |
| Montreal Cognitive Assessment (MoCA)- Beijing Version | Global cognition | Mandarin | Nasreddine et al., 2005; Yu et al., 2012 |
| Wechsler Adult Intelligence Scale-Fourth Edition (WAIS-IV) Chinese Edition | Intellectual functioning | Mandarin | Wechsler, 2008; Cui et al., 2017 |
| Wechsler Memory Scale-III (WMS-III) Chinese Edition; Spatial Span | Attention, working memory | Nonverbal (instructions in Mandarin) | Tulsky, 2003; Yao et al., 2007 |
| Boston Diagnostic Aphasia Examination (BDAE) | Language | Mandarin & English | Roth, 2011 |
| Bilingual Aphasia Test (BAT) | Language | Mandarin & English | Paradis & Libben, 1987 |
| Pyramids and Palm Trees | Semantic knowledge | Mandarin | Howard & Patterson, 1992 |
| Fuld Object Memory Evaluation (FOME) | Memory | Mandarin | Fuld, 1981 |
| Brief Visuospatial Memory Test (BVMT-R) | Memory | Nonverbal (instructions in Mandarin) | Benedict et al., 1996 |
| Color Trails Test (CTT) | Processing speed, executive functioning | Nonverbal (instructions in Mandarin) | D’Elia et al., 1996 |
| Behavioral Dyscontrol Scale | Executive functioning | Mandarin, English, nonverbal | Grigsby et al., 1992 |
| Ramparts, Spirals, Ms & Ns | Executive functioning | Nonverbal (instructions in Mandarin) | Lezak et al., 2004 |
| Grooved Pegboard | Motor functioning | Nonverbal (instructions in Mandarin) | Kløve, 1963 |
| Praxis Screen | Motor functioning | Nonverbal (instructions in Mandarin) | De Renzi et al., 1980 |

Note. Tests were administered in Mandarin, English, or nonverbally based on task demands and the patient’s language abilities. Mandarin responses were obtained with examiner interpretation when necessary. Normative data were derived from published test manuals when available; cited references reflect source materials for each instrument.
